# Surgical procedure for unexpected balloon burst complication during endoscopic balloon dilatation in a patient with common bile duct stones

**DOI:** 10.1186/s40792-020-01014-5

**Published:** 2020-10-02

**Authors:** Koji Morishita, Hideaki Sasaki

**Affiliations:** 1 Department of Acute Critical Care and Disaster Medicine, Tokyo Medical and Dental University Hospital of Medicine, 1-5-45 Yushima, Bunkyo-ku, Tokyo, 113-8510 Japan; 2 Department of Emergency Medicine, Okinawa Red Cross Hospital, Okinawa, Japan

**Keywords:** Endoscopic balloon dilatation, Common bile duct stones, Complications

## Abstract

**Background:**

Endoscopic balloon dilatation (EBD) is the established treatment for common bile duct (CBD) stones. Although pancreatitis and bleeding have been reported as major complications of EBD, balloon-related complications are rarely reported in EBD.

**Case presentation:**

A 30-year-old woman with suspected CBD stones underwent endoscopic retrograde cholangiopancreatography (ERCP) and EBD. During EBD, the balloon of the EBD catheter suddenly burst at the biliary sphincter. We therefore performed surgical intervention: removal of the broken EBD catheter and T-tube drainage. Finally, the patient was discharged without any complications.

**Conclusions:**

We present a case involving a burst balloon of an EBD catheter as a rare complication during EBD, as well as the surgical technique that was used to treat this complication.

## Background

Endoscopic balloon dilatation (EBD) of the biliary sphincter can be a valuable adjunct therapeutic option for the removal of common bile duct (CBD) stones during endoscopic retrograde cholangiopancreatography (ERCP) in selected patients [1, 2]. We herein report a very uncommon complication wherein the balloon of an EBD catheter burst during treatment of acute cholangitis.

## Case presentation

**Fig. 1:**
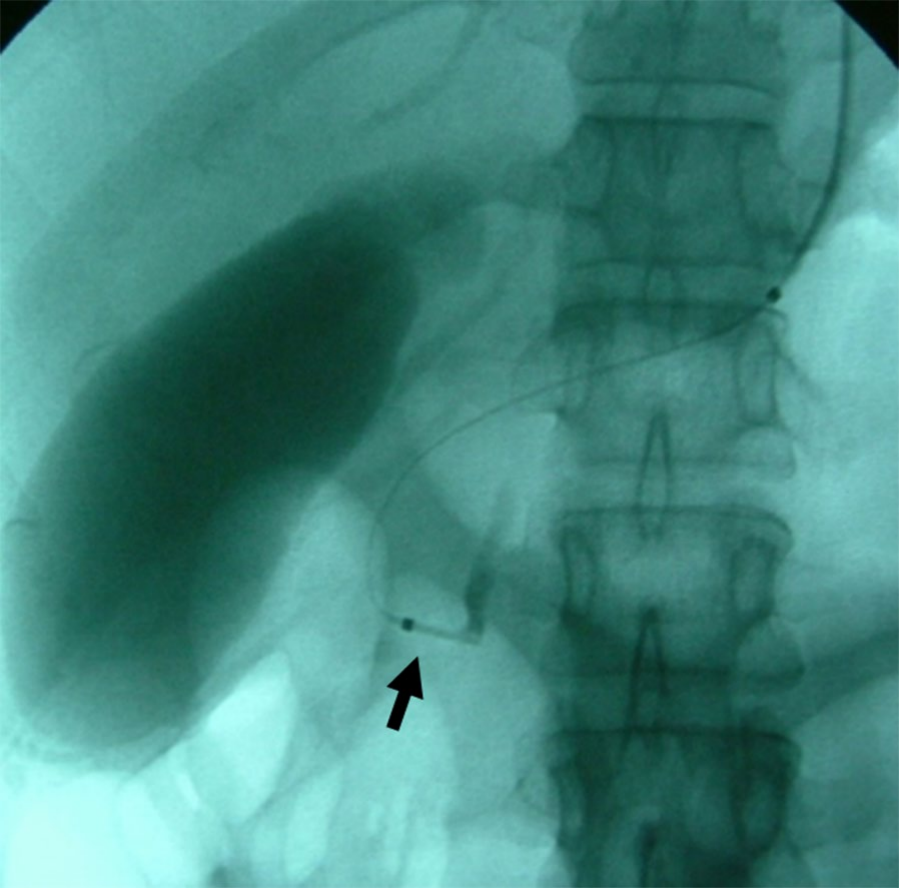
Cholangiography during endoscopic balloon dilatation (EBD). The burst balloon of the endoscopic balloon dilatation (EBD) at the site of the biliary sphincter. Arrow indicates the burst balloon of the EBD catheter at the site of the biliary sphincter

**Fig. 2:**
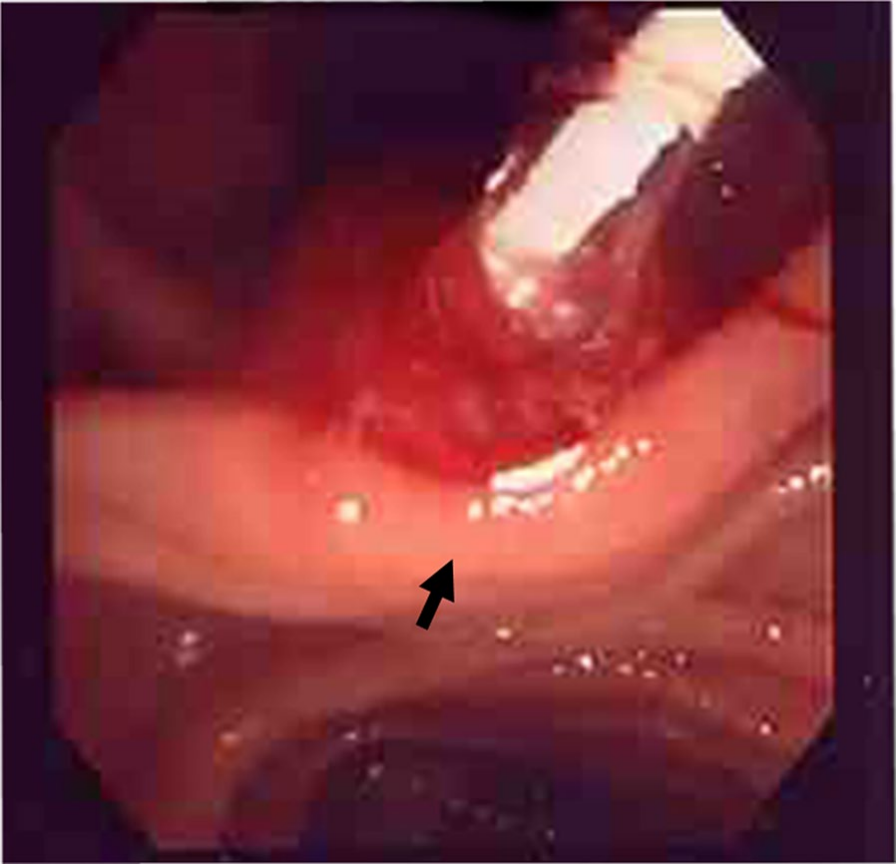
Endoscopic findings during endoscopic balloon dilatation (EBD). The burst balloon of the endoscopic balloon dilatation (EBD) at the site of the biliary sphincter. Arrow indicates the burst balloon of the EBD catheter at the site of the biliary sphincter

**Fig. 3:**
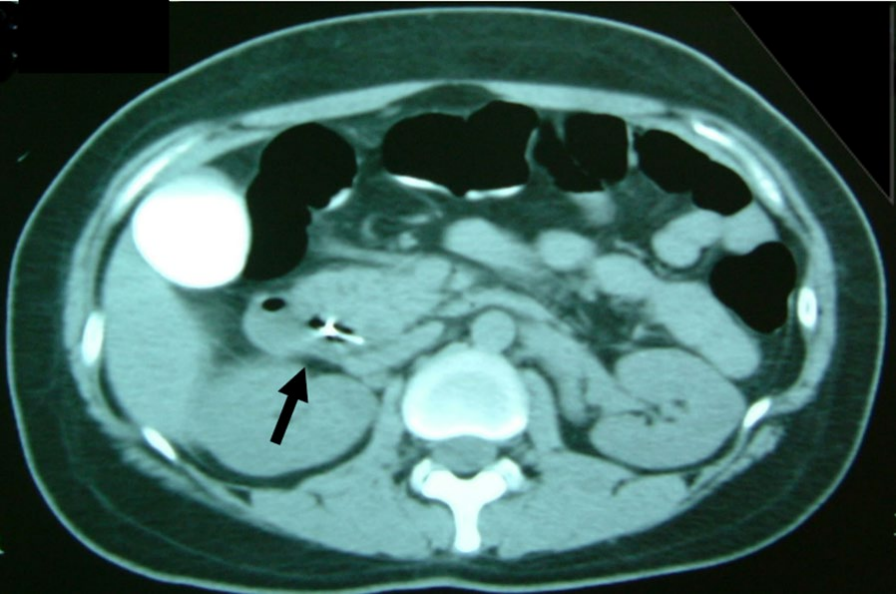
Abdominal CT. A computed tomography (CT) scan showing the burst balloon located at the site of the biliary sphincter. The arrow indicates the burst balloon of the EBD catheter at the site of the biliary sphincter

**Fig. 4:**
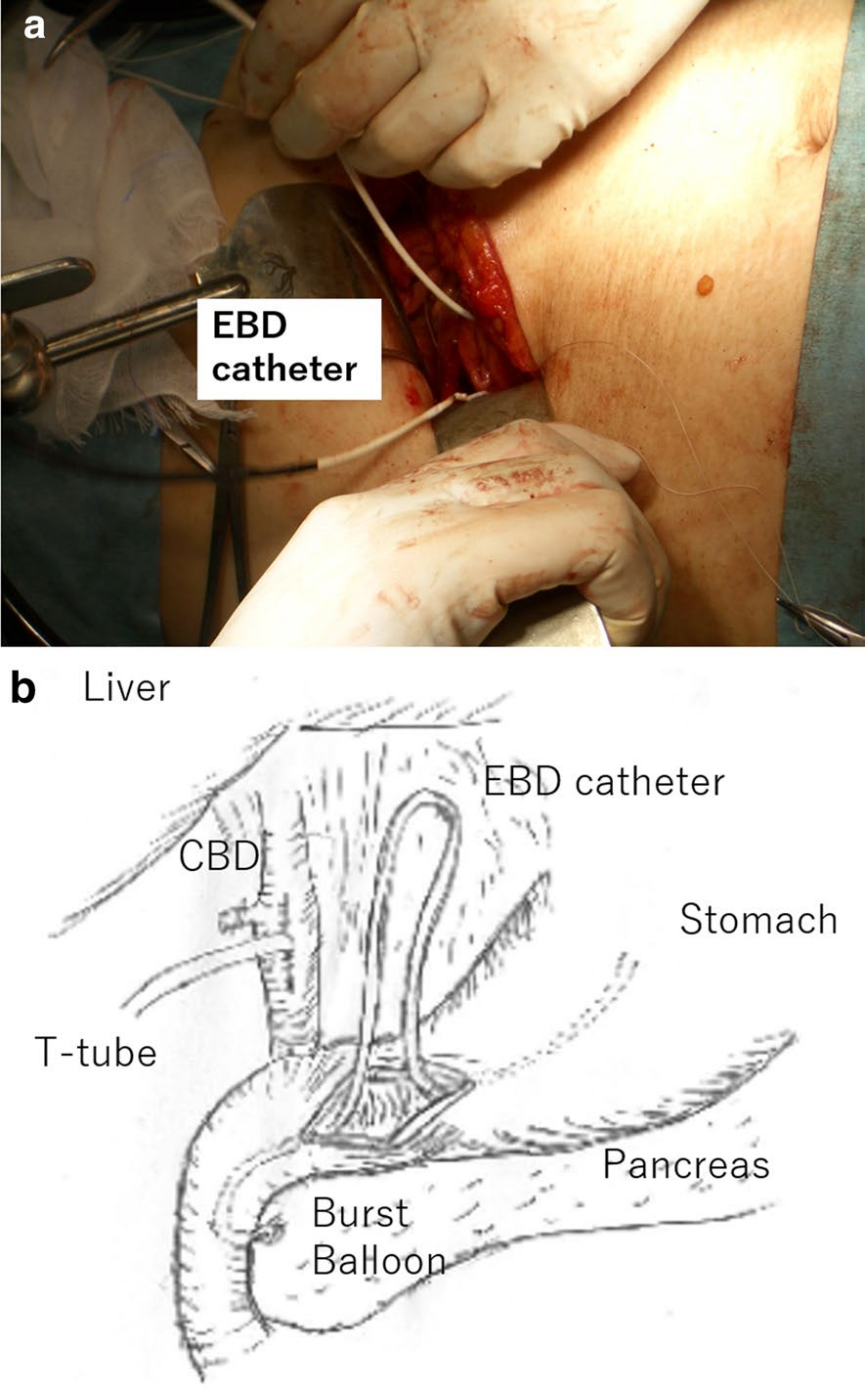
**a**, **b** The surgical findings. The burst balloon of the EBD catheter was removed during the surgical procedure. An incision at the pylorus was made to remove the EBD catheter through the incision

**Fig. 5:**
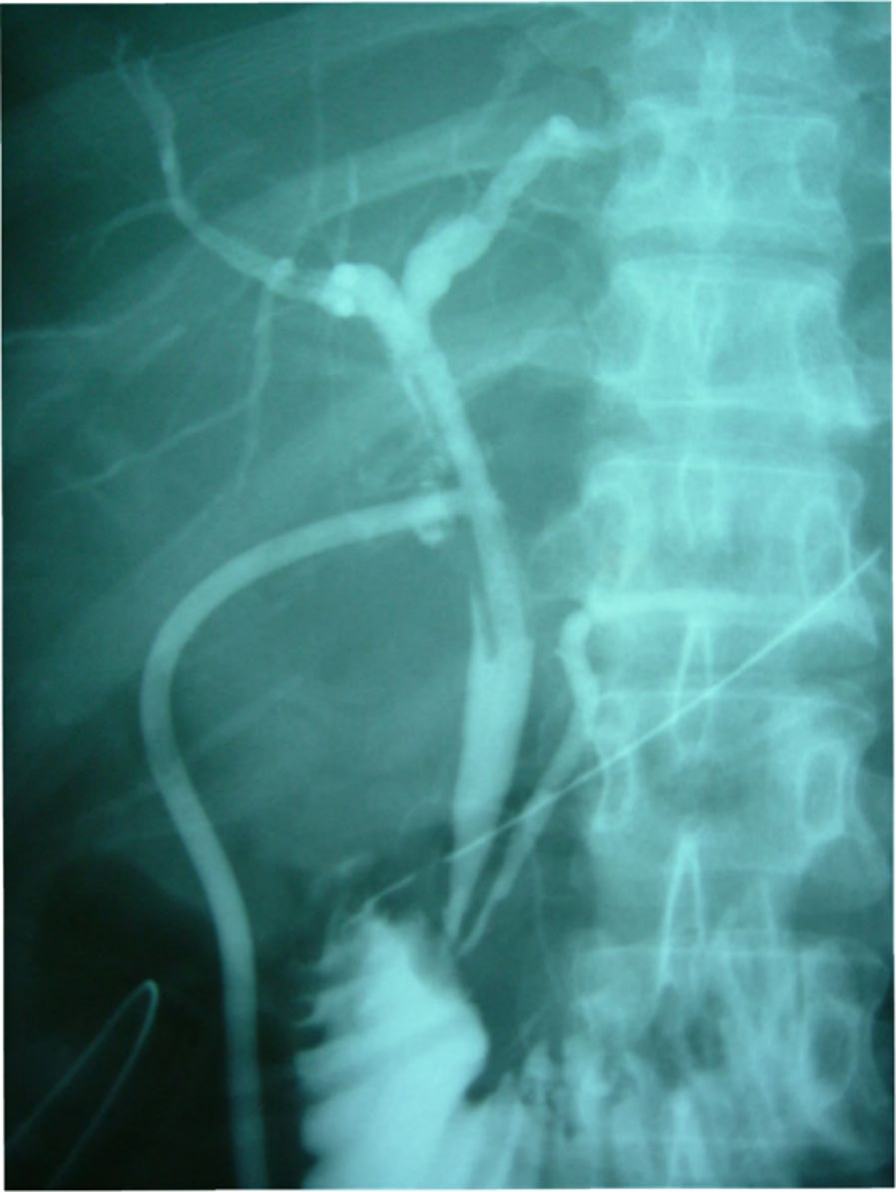
Intra-operative cholangiography. Intra-operative cholangiography revealed no residual stones or abnormalities of the biliary sphincter

**Fig. 6:**
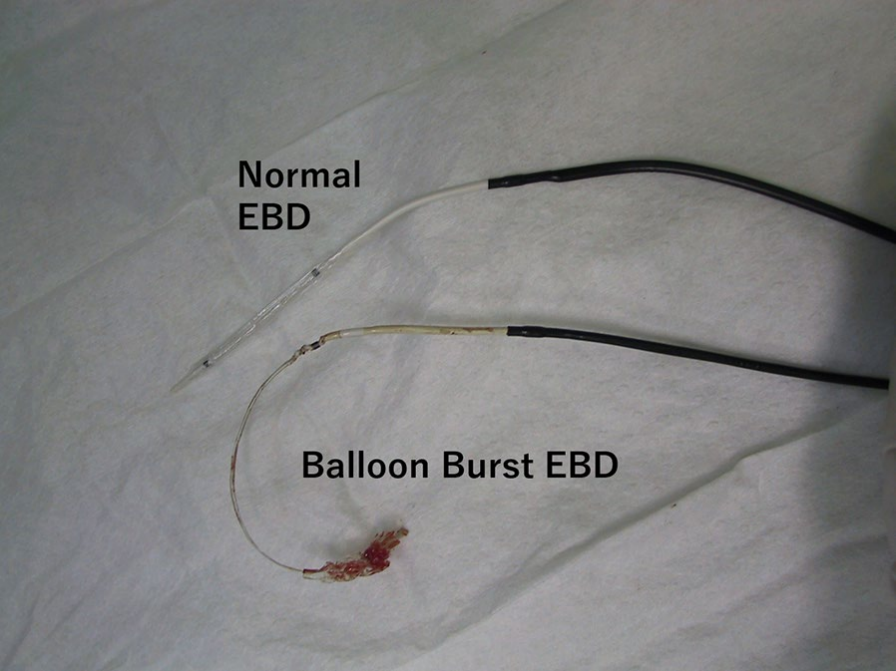
The burst balloon of the EBD catheter. The burst balloon of the EBD catheter is shown in comparison to a normal balloon of an EBD catheter

A 30-year-old woman was transported to our hospital by ambulance due to epigastric pain. A laboratory analysis revealed the following: white blood cells, 4000/mm^3^; total bilirubin, 3.0 mg/dL; alkaline phosphatase, 640 IU/L; GOT, 395 IU/L; GPT, 746 IU/L; and amylase, 37 IU/L. Abdominal CT demonstrated multiple gallbladder (GB) stones without inflammation of the GB; the diameter of the common bile duct (CBD) was 10 mm. ERCP was performed under the suspicion of CBD stones. Bile duct cannulation was easily performed. Cholangiography revealed no apparent CBD stones. Although EBD is not routinely carried out in this situation, we carefully performed EBD due to the suspicion of CBD stones, and carefully taking the clinical course into consideration. Before the procedure, a dilatation balloon (Hurricane RX Rapid Exchange, Boston Scientific, MA, USA) was inflated as a precaution in order to check the condition of the balloon. The dilatation balloon was then passed over the guidewire and located at the site of the biliary sphincter. The balloon was inflated to 2 atmospheres of pressure. After the procedure, the balloon suddenly burst. We were unable to remove the EBD catheter because the balloon was caught at the biliary sphincter (Figs. 1, 2). A computed tomography (CT) scan showing the burst balloon located at the site of the biliary sphincter (Fig. 3). Finally, we had to perform surgical intervention to remove the EBD. We made an incision at the pylorus, and then we manually pulled the EBD catheter through this incision, as shown in Fig. 4a, b. The broken catheter was successfully removed without injuring the biliary sphincter. Cholecystectomy, CBD exploration, and then removal of the CBD stone were performed. A T-tube was inserted for drainage. Intra-operative cholangiography revealed no residual stones and no biliary sphincter abnormality (Fig. 5). The burst balloon of the EBD catheter is shown in Fig. 6. The patient was discharged without any complications after removal of the T-tube on post-operative day 14.

## Discussion

EBD is the treatment of choice for patients with CBD stones. The high success rate and safety of this modality have been well established by a number of studies [1–3]. Complications of EBD, such as pancreatitis, hemorrhage, perforation, and infection have been reported [4–9]. In the current case, we demonstrated balloon burst of an EBD catheter as a rare complication of EBD. According to the product information of the EBD catheter, the balloon is made from nylon [10]. We checked the condition of balloon as a precaution; however, we could not prevent this serious complication. Although no structural abnormalities of the distal bile duct were observed in this case, stricture or a tapered distal bile duct would increase the risk of balloon-related complications. To our knowledge, the present case study represents the first report regarding a burst balloon as a complication of EBD which required surgical treatment. Through our experience, we think that it is important to provide patients with information about this complication before performing EBD. We therefore believe that we should include the information about this complication when obtaining informed consent from patients.

Regarding the surgical technique, we think that there were three important considerations in the surgical removal of the burst balloon of the EBD catheter that was trapped at the biliary sphincter. First, we made the incision at the pylorus to remove the catheter, based on the consideration that an incision at the 2nd portion of the duodenum would increase the risk of post-operative complications (e.g., duodenal stenosis or leakage). Second, we preformed cholecystectomy and intra-operative cholangiography because residual stones were present in the CBD. Third, we had to pay attention to biliary sphincter edema and dysfunction induced by the burst balloon. Thus, a T-tube was inserted into the CBD after CBD stone removal.

## Conclusion

Reports of balloon-related complications occurring during EBD are very rare. It is important to know about this serious complication and the surgical technique for that was used to treat it.

## Data Availability

The authors declare that all the data in this article are available within the article.

## Abbreviations

CBD: Common bile duct stones
ERCP: Endoscopic retrograde cholangiopancreatography
EBD: Endoscopic balloon dilatation
